# Postoperative chylothorax after left pulmonary wedge resection without mediastinal lymph node dissection: a case report

**DOI:** 10.1093/jscr/rjac634

**Published:** 2023-01-10

**Authors:** Li Chen, Bo Tian, Shaohua Xie, Xing Wei, Qiang Li, Bin Hu

**Affiliations:** Department of Thoracic Surgery, Sichuan Cancer Hospital & Institute, Sichuan Cancer Center, School of Medicine, University of Electronic Science and Technology of China, Chengdu, Sichuan, China; Department of Thoracic Surgery, Sichuan Cancer Hospital & Institute, Sichuan Cancer Center, School of Medicine, University of Electronic Science and Technology of China, Chengdu, Sichuan, China; Department of Thoracic Surgery, Sichuan Cancer Hospital & Institute, Sichuan Cancer Center, School of Medicine, University of Electronic Science and Technology of China, Chengdu, Sichuan, China; Department of Thoracic Surgery, Sichuan Cancer Hospital & Institute, Sichuan Cancer Center, School of Medicine, University of Electronic Science and Technology of China, Chengdu, Sichuan, China; Department of Thoracic Surgery, Sichuan Cancer Hospital & Institute, Sichuan Cancer Center, School of Medicine, University of Electronic Science and Technology of China, Chengdu, Sichuan, China; Department of Thoracic Surgery, Sichuan Cancer Hospital & Institute, Sichuan Cancer Center, School of Medicine, University of Electronic Science and Technology of China, Chengdu, Sichuan, China

## Abstract

Postoperative chylothorax is a rare but serious complication after pulmonary resection. In previous studies, we have found no reports of postoperative chylothorax after left pulmonary wedge resection. Considering the many variations in the route of the thoracic duct, it also has the risk of postoperative chylothorax. We describe a case of refractory chylothorax after left pulmonary wedge resection without mediastinal lymph node dissection. Conservative treatment and supradiaphragmatic thoracic duct ligation did not obtain satisfactory results in this patient. Finally, under the guidance of magnetic resonance–thoracic ductography (MRTD), we successfully ligated the thoracic duct fistula. Thus, MRTD may contribute positively to being used to locate the thoracic duct and its fistula to support precise surgical intervention.

## INTRODUCTION

A chylothorax is characterized by the accumulation of bacteriostatic chyle in the pleural space. It is primarily caused by intraoperative injury to the thoracic duct during pulmonary resection with lymph node dissection [1]. The occurrence rate of postoperative chylothorax is from 1.4 to 2.3% [2, 3]. And the occurrence rate of postoperative chylothorax in patients who underwent right-sided operations is more than in those who underwent left-sided operations [4]. There have been no reports of postoperative chylothorax after left pulmonary wedge resection without lymph node dissection in the literature. Herein, we report a case of refractory chylothorax after left pulmonary wedge resection. And it was finally resolved with twice secondary thoracic duct ligation.

## CASE

**Figure 1:**
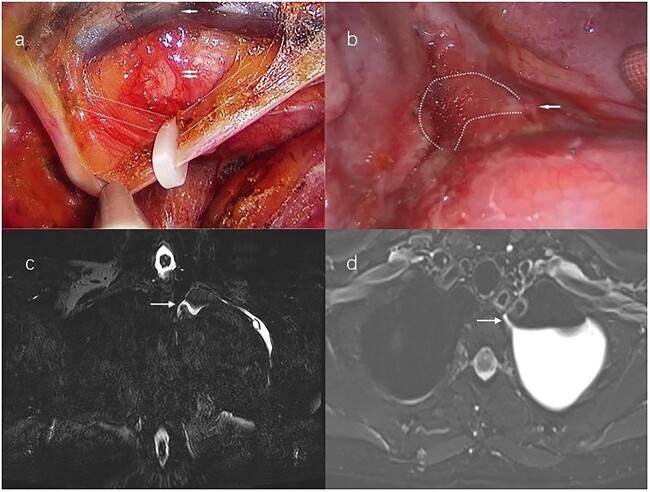
(**a**) Uniport video-assisted thoracic surgery (VATS) right supradiaphragmatic thoracic duct ligation; white broken lines mark the course of the thoracic duct; the single white arrow marks the aorta, the double white arrows mark the azygos vein. (**b**) Left lateral uniport VATS reveals that the thoracic duct fistula locates at the T2-T3 level; chylous pleural effusion flows from the fistula; the single white arrow marks the thoracic duct fistula; the white broken lines mark the chylous pleural effusion. (**c**) Magnetic resonance–thoracic discography reveals that the thoracic duct fistula is located at the left posterior part of the esophagus at the T2-T3 level; the single white arrow marks the thoracic duct fistula in the coronal view. (**d**) The thoracic duct fistula is connected to the pleural effusion in the Axial view; the single white arrow marks the thoracic duct fistula.

A 54-year-old woman without a history of smoking visited our hospital. The chest computed tomography (CT) revealed a 3.5 × 2.4 cm irregular soft tissue mass in the apicoposterior segment of the left upper lobe. All other examinations were within normal limits. For diagnosis and radical therapy, we performed left upper lobe wedge resection by uniport video-assisted thoracic surgery (VATS). Its histological finding was tuberculoma. Then we did not dissect the mediastinal lymph node. Upon beginning a regular diet postoperative day 1 (POD 1), the patient did not have any special discomfort and pleural fluid drainage was normal. But on POD 2, milky fluid was drained (700 ml/24 h) and the triglyceride level in the pleural fluid was 378 mg/dl. The patient was diagnosed with chylothorax. First, the patient received in total a series of conservative treatment measures, including total parenteral nutrition and drug therapy (somatostatin), but it had no great effect on the pleural fluid drainage volume. Until POD 7, the volume was still >500 ml/day. Therefore, she underwent conventional right supradiaphragmatic thoracic duct ligation by uniport VATS (Fig. 1a) But the condition was no improvement, and even developed high-volume, milky pleural fluid drainage >1200 ml/24 h on POD 8. Then, the patient underwent a magnetic resonance–thoracic discography (MRTD). The MRTD revealed that the thoracic duct fistula was located at the left posterior part of the esophagus at the T2–T3 level of the left chest. From the coronal view (Fig. 1c) and axial view (Fig. 1d), the relationship between the chylothorax and the thoracic duct fistula was clearly shown. Then she underwent the third operation, ligating the thoracic duct fistula by uniport VATS on POD 19 (Fig. 1b). Immediately after surgery, the fluid drainage dropped to <150 ml/day and no chyle leakage occurred. The patient gave informed consent to this study.

## DISCUSSION

Postoperative chylothorax is extremely rare after left pulmonary resections， and we have found no case reports of postoperative chylothorax after left pulmonary wedge resection. Previous studies have reported that extension of resection, right-sided surgery and systematic mediastinal lymph node dissection is the high-risk factors of chylothorax [5]. Due to the complex classification and high variation rate of the thoracic duct, even if the operation is far away from the conventional route of the thoracic duct, the branch of the thoracic duct variation may be injured, resulting in postoperative chylothorax [6]. There even have been reports of chylothorax developing after pancreatectomy [7]. The reason for the occurrence of postoperative chylothorax in this patient may be the injury of an abnormally running thoracic duct during dissociation of the upper lobe of the left lung.

For recurrent or persistent non-traumatic chylothorax, the right supradiaphragmatic thoracic duct ligation is an effective treatment [8]. It may be due to the presence of extremely rare double thoracic ducts or low hidden branches or other reasons, so the first thoracic duct ligation would not effective in our case.

With the help of MRTD, we find the thoracic duct fistula, which was the key to the success of a second thoracic duct ligation. Traditional lymphangiography uses an oil-based contrast medium, which is conventionally used to image thoracic duct configuration. It is not only technically demanding for operators but distressing for patients [6]. MRTD is a non-invasive procedure with a lower associated complication rate compared with traditional lymphangiography. We can use MRTD to guide more precisely the surgical operations and obtain a higher success rate.

## CONCLUSION

We report a case of chylothorax after wedge resection without mediastinal lymph node dissection. Due to the many variations in the route of the thoracic duct, it still has the risk of postoperative chylothorax after pulmonary wedge resection. MRTD has important value in the treatment of postoperative chylothorax. It can be used to locate the thoracic duct and its fistula to support precise surgical intervention.

## CONFLICT OF INTEREST STATEMENT

None declared.

## FUNDING

Department of Science and Technology of Sichuan Province (Grant no. 23SYSX0073) and Chengdu Science and Technology Bureau (Grant no. 2021-YF05-02138-SN).

## Supplementary Material

video_rjac634
